# Greengenes2 unifies microbial data in a single reference tree

**DOI:** 10.1038/s41587-023-01845-1

**Published:** 2023-07-27

**Authors:** Daniel McDonald, Yueyu Jiang, Metin Balaban, Kalen Cantrell, Qiyun Zhu, Antonio Gonzalez, James T. Morton, Giorgia Nicolaou, Donovan H. Parks, Søren M. Karst, Mads Albertsen, Philip Hugenholtz, Todd DeSantis, Se Jin Song, Andrew Bartko, Aki S. Havulinna, Pekka Jousilahti, Susan Cheng, Michael Inouye, Teemu Niiranen, Mohit Jain, Veikko Salomaa, Leo Lahti, Siavash Mirarab, Rob Knight

**Affiliations:** 1 Department of Pediatrics, University of California San Diego School of Medicine, La Jolla, CA USA; 2 Department of Electrical and Computer Engineering, University of California San Diego, La Jolla, CA USA; 3 Bioinformatics and Systems Biology Program, University of California San Diego, La Jolla, CA USA; 4 Department of Computer Science and Engineering, University of California San Diego, La Jolla, CA USA; 5 School of Life Sciences, Arizona State University, Tempe, AZ USA; 6 Biodesign Center for Fundamental and Applied Microbiomics, Arizona State University, Tempe, AZ USA; 7 Biostatistics & Bioinformatics Branch, Eunice Kennedy Shriver National Institute of Child Health and Human Development, National Institutes of Health, Bethesda, MD USA; 8 Halicioglu Data Science Institute, University of California San Diego, La Jolla, CA USA; 9 Australian Centre for Ecogenomics, School of Chemistry and Molecular Biosciences, The University of Queensland, St Lucia, Queensland Australia; 10 Department of Obstetrics and Gynecology, Columbia University, New York, NY USA; 11 Department of Chemistry and Bioscience, Aalborg University, Aalborg, Denmark; 12 Department of Informatics, Second Genome, Brisbane, CA USA; 13 Center for Microbiome Innovation, Jacobs School of Engineering, University of California San Diego, La Jolla, CA USA; 14 Finnish Institute for Health and Welfare, Helsinki, Finland; 15 Institute for Molecular Medicine Finland, FIMM-HiLIFE, Helsinki, Finland; 16 Division of Cardiology, Brigham and Women’s Hospital, Boston, MA USA; 17 Cedars-Sinai Medical Center, Los Angeles, CA USA; 18 Cambridge Baker Systems Genomics Initiative, Baker Heart and Diabetes Institute, Melbourne, Victoria Australia; 19 Cambridge Baker Systems Genomics Initiative, Department of Public Health and Primary Care, University of Cambridge, Cambridge, UK; 20 Division of Medicine, Turku University Hospital and University of Turku, Turku, Finland; 21 Sapient Bioanalytics, LLC, San Diego, CA USA; 22 Department of Computing, University of Turku, Turku, Finland; 23 Department of Bioengineering, University of California San Diego, La Jolla, CA USA

**Keywords:** Databases, Microbial ecology

## Abstract

Studies using 16S rRNA and shotgun metagenomics typically yield different results, usually attributed to PCR amplification biases. We introduce Greengenes2, a reference tree that unifies genomic and 16S rRNA databases in a consistent, integrated resource. By inserting sequences into a whole-genome phylogeny, we show that 16S rRNA and shotgun metagenomic data generated from the same samples agree in principal coordinates space, taxonomy and phenotype effect size when analyzed with the same tree.

## Abstract (web summary)

A comprehensive microbial resource reconciles genomic and 16S rRNA data in a single tree.

## Main

Shotgun metagenomics and 16S rRNA gene amplicon (16S) studies are widely used in microbiome research, but investigators using these different methods typically find their results hard to reconcile. This lack of standardization across methods limits the utility of the microbiome for reproducible biomarker discovery.

A key problem is that whole-genome resources and rRNA resources depend on different taxonomies and phylogenies. For example, Web of Life (WoL)^1^ and the Genome Taxonomy Database (GTDB)^2^ provide whole-genome trees that cover only a small fraction of known bacteria and archaea, while SILVA^3^ and Greengenes^4^ are more comprehensive but are most often not linked to genome records.

**Fig. 1:**
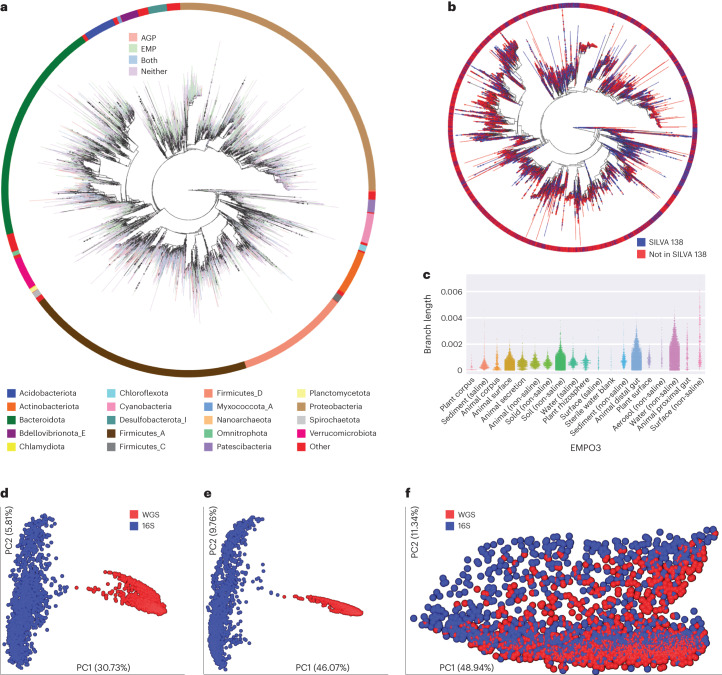
Greengenes2 overview and harmonization of 16S rRNA ASVs with shotgun metagenomic data. **a**, The Greengenes2 phylogeny rendered using Empress^23^, with ASV multifurcations collapsed; tip color indicates representation in the American Gut Project (AGP), the EMP, both or neither, with the top 20 represented phyla depicted in the outer bar. **b**, The same collapsed phylogeny colored by the presence or absence of the best BLAST^24^ hit from SILVA 138. The bar depicts the same coloring as the tips. **c**, EMP samples and the amount of novel branch length (normalized by the total backbone branch length) added to the tree through ASV fragment placement. Note that sample counts are not even across EMPO3 categories. **d**, Bray–Curtis applied to paired 16S V4 rRNA ASVs and whole-genome shotgun samples from THDMI subset of The Microsetta Initiative; PC, principal coordinate. **e**, Same data as **d** but computing Bray–Curtis on collapsed genus data. **f**, Same data as **d** and **e** but using weighted UniFrac at the ASV and genome identifier levels. Source data

We reasoned that an iterative approach could yield a single massive reference tree that unifies these different data layers (for example, genome and 16S rRNA records), which we call Greengenes2. We began with a whole-genome catalog of 15,953 bacterial and archaeal genomes that were evenly sampled from NCBI, and we reconstructed an accurate phylogenomic tree by summarizing evolutionary trajectories of 380 global marker genes using the new workflow uDance^5^. This work, namely WoL version 2 (WoL2), represents a substantial upgrade from the previously released WoL1 (10,575 genomes)^1,6^. We then added 18,356 full-length 16S rRNA sequences from the Living Tree Project (LTP) January 2022 release^7^, 1,725,274 near-complete 16S rRNA genes from Karst et al.^8^ and the Earth Microbiome Project 500 (EMP500)^9^ and all full-length 16S rRNA sequences from GTDB r207 to the genome-based backbone with uDance v1.1.0, producing a genome-supported phylogeny with 16S rRNA explicitly represented. Finally, we inserted 23,113,447 short V4 16S rRNA Deblur v1.1.0 (ref. ^10^) amplicon sequence variants (ASVs) from Qiita (retrieved 14 December 2021)^11^ and mitochondria and chloroplast 16S rRNA from SILVA v138 using deep-learning-enabled phylogenetic placement (DEPP) v0.3 (ref. ^12^). This final step represents ASVs from over 300,000 public and private samples in Qiita, including the entirety of the EMP^13^ and American Gut Project/Microsetta^14^ (Fig. 1a). Our use of uDance ensured that the genome-based relationships are kept fixed, and relationships between full-length 16S rRNA sequences are inferred. For short fragments, we kept genome and full-length relationships fixed and inserted fragments independently from each other. Following deduplication and quality control on fragment placement, this yielded a tree covering 21,074,442 sequences from 31 different EMP Ontology 3 (EMPO3) environments, of which 46.5% of species-level leaves were covered by a complete genome. Taxonomic labels were decorated onto the phylogeny using tax2tree v1.1 (ref. ^4^). The input taxonomy for decoration used GTDB r207, combined with the LTP January 2022 release. Taxonomy was harmonized prioritizing GTDB, including preserving the polyphyletic labels of GTDB (see also Methods). The taxonomy will be updated every 6 months using the latest versions of GTDB and LTP.

Greengenes2 is much larger than past resources in its phylogenetic coverage, as compared to SILVA (Fig. 1b), Greengenes (Supplementary Fig. 1a) and GTDB (Supplementary Fig. 1b). Moreover, because our amplicon library is linked to environments labeled with EMPO categories, we can easily identify the environments that contain samples that can fill out the tree. Because metagenome assembled genome (MAG) assembly efforts can only cover abundant taxa, for each EMPO category, we plotted the amount of new branch length added to the tree by taxa whose minimum abundance is 1% in each sample (Fig. 1c). The results show, on average, which environment types will best yield new MAGs and which environments harbor individual samples that will have a large impact when sequenced.

Past efforts to reconcile 16S and shotgun datasets have led to non-overlapping distributions, and only techniques such as Procrustes analysis can show relationships between the results^15^. In two large human stool cohorts^14,16^ where both 16S and shotgun data were generated on the same samples, we find that Bray–Curtis^17^ (non-phylogenetic) ordination fails to reconcile at the feature level (Fig. 1d) and is poor at the genus level (Fig. 1e and Supplementary Fig. 1c). However, UniFrac^18^, a phylogenetic method, used with our Greengenes2 tree provides better concordance (Fig. 1f and Supplementary Fig. 1d). To examine applicability of Greengenes2 to non-human environments, we next computed both Bray–Curtis and weighted UniFrac at the feature level on the 16S and shotgun data from the EMP^9^. As with the human data, we observe better concordance with the use of the Greengenes2 phylogeny (Supplementary Fig. 2) despite limited representation of whole genomes from non-human sources, as these environments are not as well characterized in general.

**Fig. 2:**
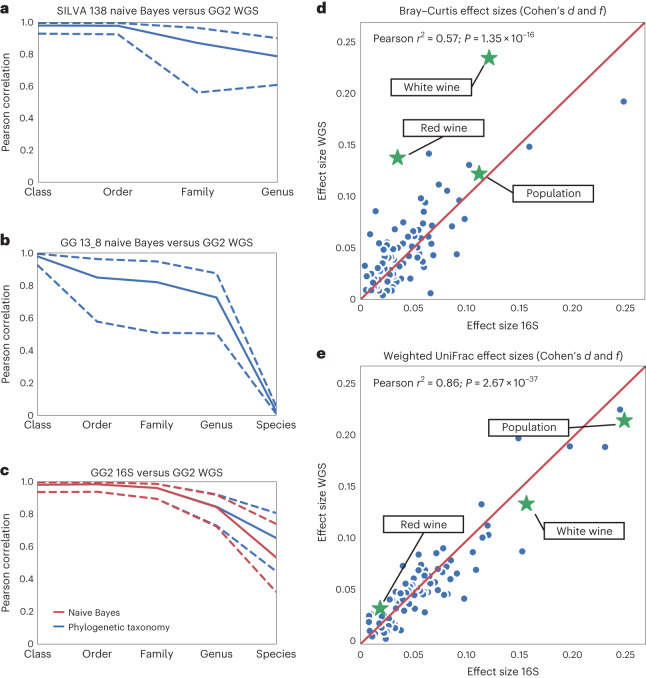
Taxonomic and effect size consistency between 16S rRNA ASVs and shotgun metagenomic data. **a**–**c**, Per-sample taxonomy comparisons between 16S and whole-genome shotgun profiles from THDMI. The solid bar depicts the 50th percentile, and the dashed lines are 25th and 75th percentiles. **a**, Assessment of 16S taxonomy with SILVA 138 using the default q2-feature-classifier naive Bayes model (note, SILVA does not annotate at the species level); GG2, Greengenes2. **b**, Assessment of 16S taxonomy with Greengenes 13_8 (GG13_8) using the default q2-feature-classifier naive Bayes model. **c**, Assessment of 16S taxonomy performed by reading the lineages directly from the phylogeny or through naive Bayes trained on the V4 regions of the Greengenes2 backbone. **d**,**e**, Effect size calculations performed with Evident on paired 16S and whole-genome shotgun samples from THDMI. Calculations were performed at maximal resolution using ASVs for 16S and genome identifiers for shotgun samples. The data represented here are human gut microbiome samples. The stars denote variables that are drawn out specifically in the plot (for example, population) and were arbitrarily selected as comparison points to help highlight differences between **d** and **e**. Bray–Curtis distances (**d**) and weighted normalized UniFrac (**e**) are shown. Source data

We also find that the per-sample shotgun and 16S taxonomy relative abundance profiles are concordant even to the species level. We first computed taxonomy profiles for shotgun data using the Woltka pipeline^19^. Using a naive Bayes classifier from q2-feature-classifier v2022.2 (ref. ^20^) to compare GTDB r207 taxonomy results at each level down to the genus level against SILVA v138 (Fig. 2a) or down to the species level against Greengenes v13_8 (Fig. 2b), no species-level reconciliation was possible. By contrast, Greengenes2 provided excellent concordance at the genus level (Pearson *r* = 0.85) and good concordance at the species level (Pearson *r* = 0.65; Fig. 2c). Interestingly, the tree is now sufficiently complete such that exact matching of 16S ASVs followed by reading the taxonomy off the tree performs even better than the naive Bayes classifier (naive Bayes, Pearson *r* = 0.54 at the species level and *r* = 0.84 at the genus level).

Finally, a critical reason to assign taxonomy is downstream use of biomarkers and indicator taxa. Microbiome science has been described as having a reproducibility crisis^21^, but much of this problem stems from incompatible methods^22^. We initially used the The Human Diet Microbiome Initiative (THDMI) dataset, which is a multipopulation expansion of The Microsetta Initiative^14^ that contains samples with paired 16S and shotgun preparations, to test whether a harmonized resource would provide concordant rankings for the variables that affect the human microbiome similarly. Using Greengenes2, the concordance was good with Bray–Curtis (Fig. 2d; Pearson *r*^2^ = 0.57), better using UniFrac with different phylogenies (SILVA 138 and Greengenes2; Supplementary Fig. 1e; Pearson *r*^2^ = 0.77) and excellent with UniFrac on the same phylogeny (Fig. 2e; Pearson *r*^2^ = 0.86). We confirmed these results with an additional cohort^16^ (Supplementary Fig. 1f,g). Intriguingly, the ranked effect sizes across different cohorts were concordant.

Taken together, these results show that use of a consistent, integrated taxonomic resource dramatically improves the reproducibility of microbiome studies using different data types and allows variables of large versus small effect to be reliably recovered in different populations.

## Methods

### Human research protocols

THDMI participant informed consent was obtained under University of California, San Diego, institutional review board protocol 141853. FINRISK participant informed consent was obtained under the Coordinating Ethical Committee of the Helsinki and Uusimaa Hospital District protocol reference number 558/E3/2001.

### Phylogeny construction

WoL2 (ref. ^1^; a tree inferred using genome-wide data) was used as the starting backbone. Full-length 16S sequences from the LTP^7^, full-length mitochondria and chloroplast from SILVA 138 (ref. ^3^), full-length 16S from GTDB r207 (ref. ^2^), full-length 16S from Karst et al.^8^ and full-length 16S from the EMP500 (ref. ^9^; samples selected and sequenced specifically for Greengenes2) were collected and deduplicated. Sequences were then aligned using UPP^25^, and gappy sequences with less than 1,000 base pairs were removed. The resulting set of 321,210 unique sequences was used with uDance v1.1.0 to update the WoL2 backbone. Briefly, uDance updates an existing tree with new sequences and (unlike placement methods) also infers the relationship of existing sequences. uDance has two modes, one that allows updates to the backbone and one that keeps the backbone fixed, where the former mode is intended for use with whole genomes. In our analyses, we kept the backbone tree (inferred using genomic data) fixed. To extend the genomic tree with 16S data, we identified 13,249 (of 15,953 total) genomes in the WoL2 backbone tree with at least one 16S copy and used them to train a DEPP model with the weighted average method detailed later to handle multiple copies. We then used DEPP to insert all 16S copies of all genomes into the backbone and measured the distance between the genome position and the 16S position. We removed copies that were placed much further than others, as identified using a two-means approach with centroids equal to at least 13 branches. We repeated this process in a second round. For every remaining genome, we selected as its representative the copy with the minimum placement error and computed the consensus with ties. At the end, we were left with 12,344 unique 16S sequences across all WoL2 genomes. For tree inference, uDance used IQ-TREE2 (ref. ^26^) in fast tree search with model GTR+ Γ after removing duplicate sequences.

Next, we collected 16S V4 ASVs from Qiita^11^ using redbiom^27^ (query performed 14 December 2021) from contexts ‘Deblur_2021.09-Illumina-16S-V4-90nt-dd6875’, ‘Deblur_2021.09-Illumina-16S-V4-100nt-50b3a2’, ‘Deblur_2021.09-Illumina-16S-V4-125nt-92f954’, ‘Deblur_2021.09-Illumina-16S-V4-150nt-ac8c0b’, ‘Deblur_2021.09-Illumina-16S-V4-200nt-0b8b48’ and ‘Deblur_2021.09-Illumina-16S-V4-250nt-8b2bff’ and aligned them to the existing 16S alignment of sequences in WoL2 using UPP, setting the maximum alignment subset size to 200 (to help with scalability). The collected 16S V4 ASVs are aligned to the V4 region of the existing ‘backbone’ alignments. A DEPP model was then trained on the full-length 16S sequences from the backbone. DEPP constructs a neural network model that embeds sequences in high-dimensional spaces such that embedded points resemble the phylogeny in their distances. Such a model then allows insertion of new sequences into a tree using the distance-based phylogenetic insertion method APPLES-2 (ref. ^28^). The ASVs from redbiom were then inserted into the backbone using the trained DEPP model. To enable analyses of large datasets, we used a clustering approach with DEPP. We trained an ensemble of DEPP models corresponding to different parts of the tree and used a classifier to detect the correct subtree. During training, for species with multiple 16S, all the copies are mapped to the same leaf in the backbone tree. To train the DEPP models with multiple sequences mapped to a leaf, each site in each sequence is encoded as a probability vector of four nucleotides across all the copies.

### Integrating the GTDB and LTP taxonomies

GTDB and LTP are not directly compatible due to differences in their curation. As a result, it is not always possible to map a species from one resource to the other because parts of a species lineage are not present, are described using different names or have an ambiguous association due to polyphyletic taxa in GTDB (for example, Firmicutes_A, Firmicutes_B and so on; https://gtdb.ecogenomic.org/faq#why-do-some-family-and-higher-rank-names-end-with-an-alphabetic-suffix). We integrated taxonomic data from LTP into GTDB as LTP includes species that are not yet represented in GTDB. Additionally, GTDB is actively curated, while LTP generally uses the NCBI taxonomy. To account for these differences, we first mapped any species that had a perfect species name association and revised its ancestral lineage to match GTDB. Next, we generated lineage rewrite rules using the GTDB record metadata. Specifically, we limited the metadata to records that are GTDB representatives and NCBI-type material and defined a lineage renaming from the recorded NCBI taxonomy to the GTDB taxonomy. These rewrite rules were applied from most- to least-specific taxa, and through this mechanism, we could revise much of the higher ranks of LTP. We then identified incertae sedis records in LTP that we could not map, removed their lineage strings and did not attempt to provide taxonomy for them, instead opting to rely on downstream taxonomy decoration to resolve their lineages. Next, any record that was ambiguous to map was split into a secondary taxonomy for use in backfilling in the downstream taxonomy decoration. Finally, we instrumented numerous consistency checks in the taxonomy through the process to capture inconsistent parents in the taxonomic hierarchy and consistent numbers of ranks in a lineage and to ensure that the resulting taxonomy was a strict hierarchy.

### Taxonomy decoration

The original tax2tree algorithm was not well suited for a large volume of species-level records in the backbone, as the algorithm requires an internal node to place a name. If two species are siblings, the tree would lack a node to contain the species label for both taxa. To account for this, we updated the algorithm to insert ‘placeholder’ nodes with zero branch length as the parents of backbone records, which could accept these species labels. We further updated tax2tree to operate directly on .jplace data^29^, preserving edge numbering of the original edges before adding ‘placeholder’ nodes. To support LTP records that could not be integrated into GTDB, we instrumented a secondary taxonomy mode for tax2tree. Specifically, following the standard decoration, backfilling and name promotion procedures, we determine on a per-record basis for the secondary taxonomy what portion of the lineage is missing and place the missing labels on the placeholder node. We then issue a second round of name promotion using the existing tax2tree methods.

The actual taxonomy decoration occurs on the backbone tree, which contains only full-length 16S records and does not contain ASVs. This is done as ASV placements are independent, do not modify the backbone and would substantially increase the computational resources required. After the backbone is decorated, fragment placements from DEPP are resolved using a multifurcation strategy using the balanced-parentheses library^30^.

### Phylogenetic collapse for visualization

We are unaware of phylogenetic visualization software that can display a tree with over 20,000,000 tips. To produce the visualizations in Fig. 1, we reduced the dimension of the tree by collapsing fragment multifurcations to single nodes, dropping the tree to 522,849 tips.

### MAG target environments

A feature table for the 27,015 16S rRNA V4 90-nucleotide EMP samples was obtained from redbiom. The ASVs were filtered to the overlap of ASVs present in Greengenes2. Any feature with <1% relative abundance within a sample was removed. The feature table was then rarefied to 1,000 sequences per sample. The amount of novel branch length was then computed per sample by summing the branch length of each ASV’s placement edge. The per-sample branch length was then normalized by the total tree branch length (excluding length contributed by ASVs).

### Per-sample taxonomy correlations

All comparisons used THDMI^14^ 16S and Woltka processed shotgun data. These data were accessed from Qiita study 10317 and filtered the set of features that overlap with Greengenes2 using the QIIME 2 (ref. ^31^) q2-greengenes2 plugin. The 16S taxonomy was assessed using either a traditional naive Bayes classifier with q2-feature-classifier and default references from QIIME 2 2022.2 or by reading the lineage directly from the phylogeny. To help improve correlations between SILVA and Greengenes2 and between Greengenes and Greengenes2, we stripped polyphyletic labelings from those data; we did not strip polyphyletic labels from the phylogenetic taxonomy comparison or the Greengenes2 16S versus Greengenes2 whole-genome shotgun (WGS) naive Bayes comparison. Shotgun taxonomy was determined by the specific observed genome records. Once the 16S taxonomy was assigned, those tables and the WGS Woltka WoL2 table were collapsed at the species, genus, family, order and class levels. We then computed a minimum relative abundance per sample in the dataset from THDMI. In each sample, we removed any feature, either 16S or WGS, below the per-sample minimum (that is, max(min(16S), min(WGS))), forming a common minimal basis for taxonomy comparison. Following filtering, Pearson correlation was computed per sample using SciPy^32^. These correlations were aggregated per 16S taxonomy assignment method and by each taxonomic rank. The 25th, 50th and 75th percentiles were then plotted with Matplotlib^33^.

### Principal coordinates

THDMI Deblur 16S and Woltka processed shotgun sequencing data, against WoL2, were obtained from Qiita study 10317. Both feature tables were filtered against Greengenes2 2022.10, removing any feature not present in the tree. For the genus collapsed plot (Fig. 1e), both the 16S and WGS data features were collapsed using the same taxonomy. For all three figures, the 16S data were subsampled, with replacement, to 10,000 sequences per sample. The WGS data were subsampled, with replacement, to 1,000,000 sequences per sample. Bray–Curtis, weighted UniFrac and principal coordinates analysis were computed using q2-diversity 2022.2. The resulting coordinates were visualized with q2-emperor^34^.

The EMP ‘EMP500’ 16S and Woltka processed shotgun sequencing data, against WoL2, were obtained from Qiita study 13114. Both feature tables were filtered against Greengenes2 2022.10. The 16S data were subsampled, with replacement, to 1,000 sequences per sample. The WGS data were subsampled, with replacement, to 50,000 sequences per sample. The sequencing depth for WGS data was selected based on Supplementary Fig. 6 of Shaffer et al.^9^, which noted low levels of read recruitment to publicly available whole genomes. Bray–Curtis, weighted UniFrac and principal coordinates analysis were computed using q2-diversity 2022.2. The resulting coordinates were visualized with q2-emperor.

### Effect size calculations

Similar to principal coordinates, data from THDMI were rarefied to 9,000 and 2,000,000 sequences per sample for 16S and WGS, respectively. Bray–Curtis and weighted normalized UniFrac were computed on both sets of data. The variables for THDMI were subset to those with at least two category values having more than 50 samples. For UniFrac with SILVA (Supplementary Fig. 1e), we performed fragment insertion using q2-fragment-insertion^35^ into the standard QIIME 2 SILVA reference, followed by rarefaction to 9,000 sequences per sample, and then computed weighted normalized UniFrac.

For FINRISK, the data were rarefied to 1,000 and 500,000 sequences per sample for 16S and WGS, respectively. A different depth was used to account for the overall lower amount of sequencing data for FINRISK. As with THDMI, the variables selected were reduced to those with at least two category values having more than 50 samples.

Support for computing paired effect sizes is part of the QIIME2 Greengenes2 plugin q2-greengenes2, which performs effect size calculations using Evident^36^.

### Reporting summary

Further information on research design is available in the Nature Portfolio Reporting Summary linked to this article.

## Online content

Any methods, additional references, Nature Portfolio reporting summaries, source data, extended data, supplementary information, acknowledgements, peer review information; details of author contributions and competing interests; and statements of data and code availability are available at 10.1038/s41587-023-01845-1.

### Supplementary information

Supplementary Information Supplementary Figs. 1 and 2.

Reporting Summary

Supplementary Data 1 Data points for Supplementary Fig. 2a,b.

### Source data

Source Data Fig. 1 Data points for the plots in Fig. 1, where appropriate.

Source Data Fig. 2 Data points for the plots in Fig. 2.

## Data Availability

The official location of the Greengenes2 releases is http://ftp.microbio.me/greengenes_release/. The data are released under a BSD-3 clause license. Data from THDMI are part of Qiita study 10317 and European Bioinformatics Institute accession number PRJEB11419. The FINRISK data and including the data presented in Supplementary Fig. 1c–g are protected; details on data access are available in the European Genome–Phenome Archive under accession number EGAD00001007035. The data presented in Supplementary Fig. 1a,b are not compatible with Excel. The EMP data are part of Qiita study 13114 and European Bioinformatics Institute accession number ERP125879. Source data are provided with this paper.

## Code availability

A QIIME 2 plugin is available to facilitate use with the resource that can be obtained from ref. ^37^ (version 2023.3; 10.5281/zenodo.7758134). Taxonomy construction, decoration and release processing is part of ref. ^38^ (version 2023.3; 10.5281/zenodo.7758138). uDance is available at GitHub^39^ (version v1.1.0; 10.5281/zenodo.7758289). Phylogeny insertion using DEPP is available at ref. ^40^ (version 0.3; 10.5281/zenodo.7768798). The trained model can be accessed via Zenodo at 10.5281/zenodo.7416684. Code used for the figures in this manuscript is available in ref. ^41^. Finally, an interactive website to explore the Greengenes2 data is available at https://greengenes2.ucsd.edu.
