# Author Correction: Greengenes2 unifies microbial data in a single reference tree

**DOI:** 10.1038/s41587-023-02026-w

**Published:** 2023-10-18

**Authors:** Daniel McDonald, Yueyu Jiang, Metin Balaban, Kalen Cantrell, Qiyun Zhu, Antonio Gonzalez, James T. Morton, Giorgia Nicolaou, Donovan H. Parks, Søren M. Karst, Mads Albertsen, Philip Hugenholtz, Todd DeSantis, Se Jin Song, Andrew Bartko, Aki S. Havulinna, Pekka Jousilahti, Susan Cheng, Michael Inouye, Teemu Niiranen, Mohit Jain, Veikko Salomaa, Leo Lahti, Siavash Mirarab, Rob Knight

**Affiliations:** 1 Department of Pediatrics, University of California San Diego School of Medicine, La Jolla, CA USA; 2 Department of Electrical and Computer Engineering, University of California San Diego, La Jolla, CA USA; 3 Bioinformatics and Systems Biology Program, University of California San Diego, La Jolla, CA USA; 4 Department of Computer Science and Engineering, University of California San Diego, La Jolla, CA USA; 5 School of Life Sciences, Arizona State University, Tempe, AZ USA; 6 Biodesign Center for Fundamental and Applied Microbiomics, Arizona State University, Tempe, AZ USA; 7 Biostatistics & Bioinformatics Branch, Eunice Kennedy Shriver National Institute of Child Health and Human Development, National Institutes of Health, Bethesda, MD USA; 8 Halicioglu Data Science Institute, University of California San Diego, La Jolla, CA USA; 9 Australian Centre for Ecogenomics, School of Chemistry and Molecular Biosciences, The University of Queensland, St Lucia, Queensland Australia; 10 Department of Obstetrics and Gynecology, Columbia University, New York, NY USA; 11 Department of Chemistry and Bioscience, Aalborg University, Aalborg, Denmark; 12 Department of Informatics, Second Genome, Brisbane, CA USA; 13 Center for Microbiome Innovation, Jacobs School of Engineering, University of California San Diego, La Jolla, CA USA; 14 Finnish Institute for Health and Welfare, Helsinki, Finland; 15 Institute for Molecular Medicine Finland, FIMM-HiLIFE, Helsinki, Finland; 16 Division of Cardiology, Brigham and Women’s Hospital, Boston, MA USA; 17 Cedars-Sinai Medical Center, Los Angeles, CA USA; 18 Cambridge Baker Systems Genomics Initiative, Baker Heart and Diabetes Institute, Melbourne, Victoria Australia; 19 Cambridge Baker Systems Genomics Initiative, Department of Public Health and Primary Care, University of Cambridge, Cambridge, UK; 20 Division of Medicine, Turku University Hospital and University of Turku, Turku, Finland; 21 Sapient Bioanalytics, LLC, San Diego, CA USA; 22 Department of Computing, University of Turku, Turku, Finland; 23 Department of Bioengineering, University of California San Diego, La Jolla, CA USA

**Keywords:** Databases, Microbial ecology

Correction to: *Nature Biotechnology* 10.1038/s41587-023-01845-1, published online 27 July 2023.

In the version of the article initially published, a portion of the Competing interests statement was missing: “D.M. is a consultant for BiomeSense, Inc., has equity and receives income. The terms of these arrangements have been reviewed and approved by the University of California, San Diego in accordance with its conflict of interest policies. R.K. is a scientific advisory board member, and consultant for BiomeSense, Inc., has equity and receives income. He is a scientific advisory board member and has equity in GenCirq. He is a consultant and scientific advisory board member for DayTwo, and receives income. He has equity in and acts as a consultant for Cybele. He is a co-founder of Biota, Inc., and has equity. He is a cofounder of Micronoma, and has equity and is a scientific advisory board member. The terms of this arrangement have been reviewed and approved by the University of California, San Diego in accordance with its conflict of interest policies.” The text has been inserted in the HTML and PDF versions of the article.

