# Supplementary material for: Greengenes2 unifies microbial data in a single reference tree
Source: Nat Biotechnol. 2023 Jul 27;42(5):715–8. doi: 10.1038/s41587-023-01845-1 (PMC10818020; doi:10.1038/s41587-023-01845-1)
Supplement: Supplementary file 1 — Supplementary Figs. 1 and 2. [file 41587_2023_1845_MOESM1_ESM.pdf]

nature biotechnology

Brief Communication

https://doi.org/10.1038/s41587-023-01845-1

Greengenes2 unifies microbial data in a single reference tree

In the format provided by the authors and unedited

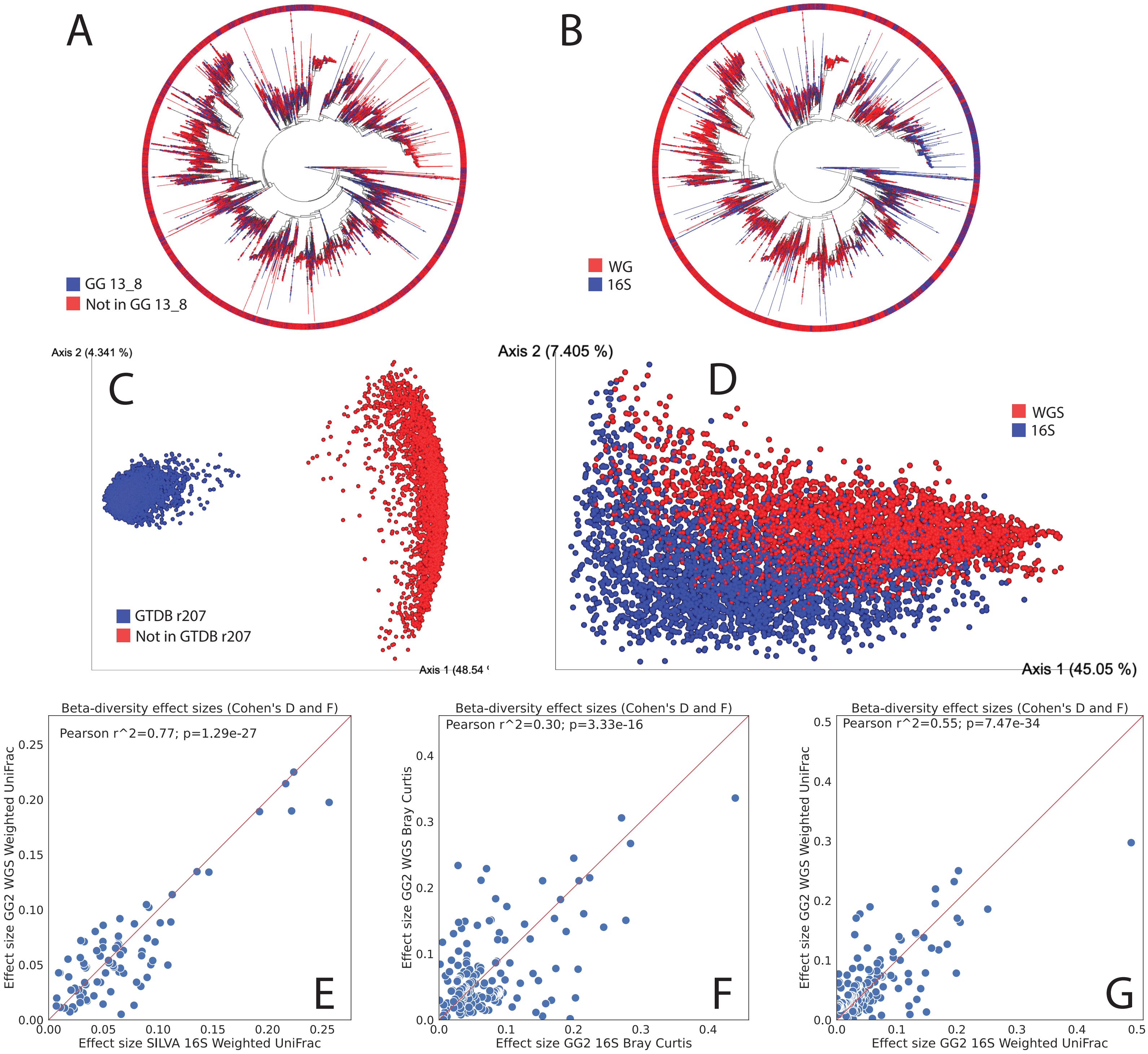

Figure S1. (A) Best BLAST hit for Greengenes 13_8 99% OTUs against Greengenes2. (B) Best BLAST hit for GTDB r207 SSU sequences against Greengenes2. (C) The FINRISK 16S and WGS data combined, collapsed to genus relative to Greengenes2, with Bray-Curtis computed followed by Principal Coordinates Analysis, colored by technical preparation. (D) The same data as (C) but using weighted UniFrac. (E) Effect sizes of the THDMI data using the SILVA 138 phylogeny for 16S data, and the Greengenes2 phylogeny for WGS data. (F) Effect sizes of the FINRISK data using Bray-Curtis. (G) The same data as (E) but using Weighted UniFrac.

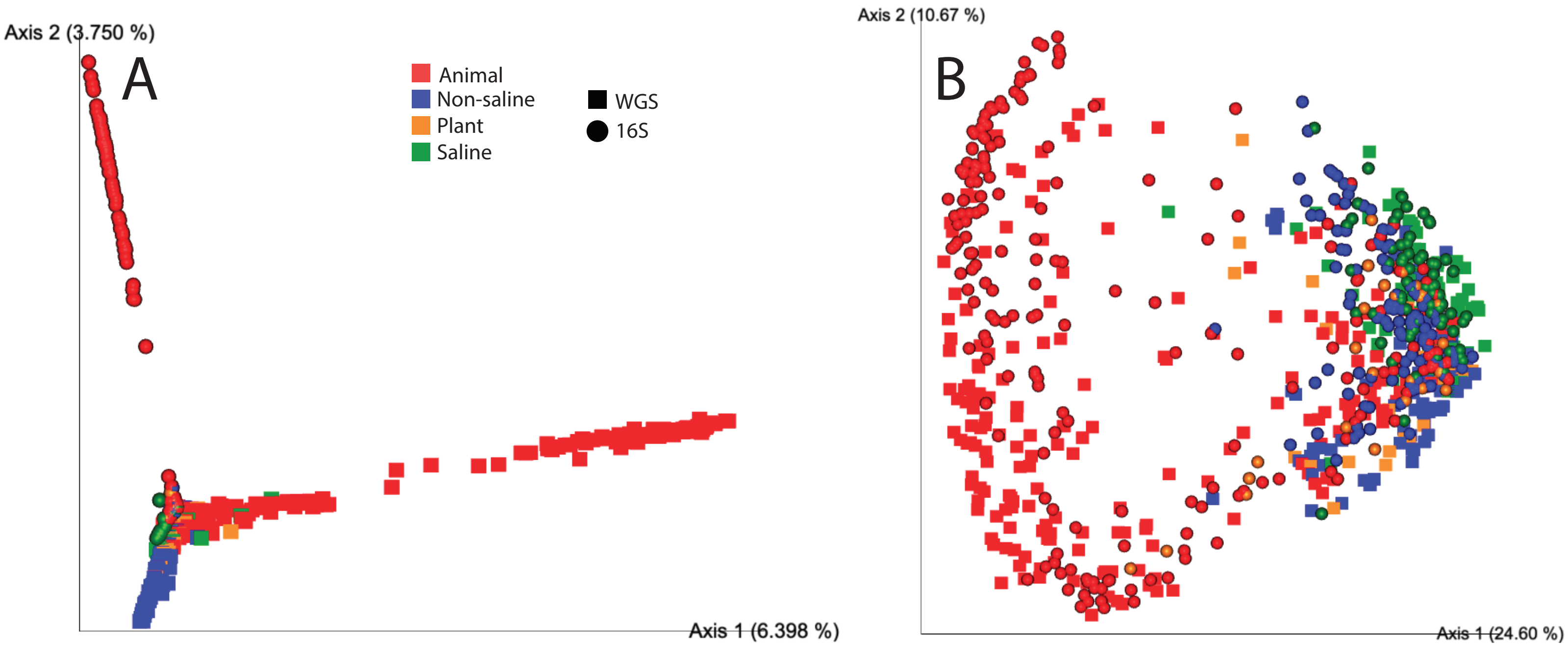

Figure S2. (A) The Earth Microbiome Project 16S and WGS data combined, and with Bray-Curtis computed at the feature level followed by Principal Coordinates Analysis, colored by Earth Microbiome Project Ontology level 2. Squares represent WGS data, and spheres represent 16S data. (B) The same data and coloring as in (A) but using weighted UniFrac.
