# Supplementary material for: Greengenes2 unifies microbial data in a single reference tree
Source: Nat Biotechnol. 2023 Jul 27;42(5):715–8. doi: 10.1038/s41587-023-01845-1 (PMC10818020; doi:10.1038/s41587-023-01845-1)
Supplement: Supplementary file 2 — Reporting Summary [file 41587_2023_1845_MOESM2_ESM.pdf]

Corresponding author(s): Rob Knight
Last updated by author(s): Apr 25, 2023

Reporting Summary

Nature Portfolio wishes to improve the reproducibility of the work that we publish. This form provides structure for consistency and transparency in reporting. For further information on Nature Portfolio policies, see our Editorial Policies and the Editorial Policy Checklist.

Statistics

For all statistical analyses, confirm that the following items are present in the figure legend, table legend, main text, or Methods section.

n/a	Confirmed	
☐	☐	The exact sample size (*n*) for each experimental group/condition, given as a discrete number and unit of measurement
☒	☐	A statement on whether measurements were taken from distinct samples or whether the same sample was measured repeatedly
☐	☒	The statistical test(s) used AND whether they are one- or two-sided<br>*Only common tests should be described solely by name; describe more complex techniques in the Methods section.*
☐	☒	A description of all covariates tested
☐	☒	A description of any assumptions or corrections, such as tests of normality and adjustment for multiple comparisons
☒	☐	A full description of the statistical parameters including central tendency (e.g. means) or other basic estimates (e.g. regression coefficient) AND variation (e.g. standard deviation) or associated estimates of uncertainty (e.g. confidence intervals)
☐	☒	For null hypothesis testing, the test statistic (e.g. *F*, *t*, *r*) with confidence intervals, effect sizes, degrees of freedom and *P* value noted<br>*Give P values as exact values whenever suitable.*
☒	☐	For Bayesian analysis, information on the choice of priors and Markov chain Monte Carlo settings
☒	☐	For hierarchical and complex designs, identification of the appropriate level for tests and full reporting of outcomes
☐	☒	Estimates of effect sizes (e.g. Cohen's *d*, Pearson's *r*), indicating how they were calculated

Our web collection on statistics for biologists contains articles on many of the points above.

Software and code

Policy information about availability of computer code

Data collection	Full length 16S operons were collected from Qiita (https://qiita.ucsd.edu)<br>THDMI, EMP and FINRISK data were collected from Qiita (https://qiita.ucsd.edu)<br>Amplicon sequence variants were collected from redbiom (v0.3.7)<br>GTDB r207 SSU sequences were obtained from their FTP<br>SILVA 138 sequences were obtained from their FTP<br>The LTP 01.2022 sequences and taxonomy were obtained from their FTP<br>Web of Life 2 was obtained directly, these data are now available by FTP (http://ftp.microbio.me/pub/wol2/)
Data analysis	Figure 1A used a multifurcation collapse, implemented in q2-greengenes2 (v2022.10; https://github.com/biocore/q2-greengenes2), and Empress (v1.2.0; https://github.com/biocore/empress) for visualization.<br>Figure 1B, S1A-B used the same multifurcation collapse in 1A, and also used BLAST 2.12.0<br>Figure 1C used custom code, available under (https://github.com/knightlab-analyses/greengenes2)<br>Figures 1D-F, S1C-D, S2 used QIIME 2 2022.11 q2-diversity and q2-emperor. 1E, S1C-D also used q2-taxa<br>Figure 2A-C used custom code, available under (https://github.com/knightlab-analyses/greengenes2)<br>Figure 2D-E, S1E-G used custom code now part of q2-greengenes2

For manuscripts utilizing custom algorithms or software that are central to the research but not yet described in published literature, software must be made available to editors and reviewers. We strongly encourage code deposition in a community repository (e.g. GitHub). See the Nature Portfolio guidelines for submitting code & software for further information.

Data

Policy information about availability of data

All manuscripts must include a data availability statement. This statement should provide the following information, where applicable:

- Accession codes, unique identifiers, or web links for publicly available datasets
- A description of any restrictions on data availability
- For clinical datasets or third party data, please ensure that the statement adheres to our policy

The official location of the Greengenes2 releases is http://ftp.microbio.me/greengenes_release/. The data are released under a BSD-3 clause license. A QIIME 2 plugin is available to facilitate use with the resource that can be obtained from https://github.com/biocore/q2-greengenes2/ (version 2023.3; DOI: 10.5281/zenodo.7758134). Taxonomy construction, decoration, and release processing is part of https://github.com/biocore/greengenes2 (version 2023.3; DOI: 10.5281/zenodo.7758138). uDance is available at GitHub: https://github.com/balabanmetin/uDance (version v1.1.0; DOI: 10.5281/zenodo.7758289). Phylogeny insertion using DEPP is available at https://github.com/yueyujiang/DEPP (version 0.3; DOI: 10.5281/zenodo.7768798); the trained model accessioned with Zenodo at 10.5281/zenodo.7416684. The THDMI data are part of Qiita study 10317, and EBI accession PRJEB11419. The FINRISK data are available under EGAD00001007035. Finally, an interactive website to explore the Greengenes2 data is available at https://greengenes2.ucsd.edu.

Research involving human participants, their data, or biological material

Policy information about studies with human participants or human data. See also policy information about sex, gender (identity/presentation), and sexual orientation and race, ethnicity and racism.

Reporting on sex and gender	The examination of human data was for technical consistency between two different types of sequence preparations. The focus of analyses in this manuscript was not on specific data associated with human participants.<br><br>Neither sex nor gender was considered in the effect size correlations of the THDMI data, the exclusion was unintentional. Sex was included in effect size correlations with the FINRISK data but not examined specifically.
Reporting on race, ethnicity, or other socially relevant groupings	We used a socially constructed variable, THDMI_cohort, to denote what country participants of THDMI took part from.
Population characteristics	n/a
Recruitment	Participants in THDMI were recruited primarily through social media. There is likely a self selection bias for those interested in their own diets. FINRISK recruitment is described at https://thl.fi/en/web/thlfi-en/research-and-development/research-and-projects/the-national-finrisk-study
Ethics oversight	Participants in THDMI are part of the American Gut Project covered by UC San Diego HRPP protocol 141853. Details on the FINRISK ethical oversight are outlined in https://academic.oup.com/ije/article/47/3/696/4641873

Note that full information on the approval of the study protocol must also be provided in the manuscript.

Field-specific reporting

Please select the one below that is the best fit for your research. If you are not sure, read the appropriate sections before making your selection.

☒ Life sciences ☐ Behavioural & social sciences ☐ Ecological, evolutionary & environmental sciences

For a reference copy of the document with all sections, see nature.com/documents/nr-reporting-summary-flat.pdf

Life sciences study design

All studies must disclose on these points even when the disclosure is negative.

Sample size	We used all available paired 16S and WGS samples from the THDMI, EMP500 and FINRISK datasets.
Data exclusions	n/a
Replication	We demonstrate an ability to integrate 16S and WGS datasets using two independent human sample sets, as well as with environmental samples.
Randomization	n/a
Blinding	n/a

Reporting for specific materials, systems and methods

We require information from authors about some types of materials, experimental systems and methods used in many studies. Here, indicate whether each material, system or method listed is relevant to your study. If you are not sure if a list item applies to your research, read the appropriate section before selecting a response.

Materials & experimental systems

n/a	Involved in the study
☒	☐ Antibodies
☒	☐ Eukaryotic cell lines
☒	☐ Palaeontology and archaeology
☒	☐ Animals and other organisms
☒	☐ Clinical data
☒	☐ Dual use research of concern
☒	☐ Plants

Methods

n/a	Involved in the study
☒	☐ ChIP-seq
☒	☐ Flow cytometry
☒	☐ MRI-based neuroimaging
